# Selective digestive decontamination

**DOI:** 10.1186/s13613-025-01546-9

**Published:** 2025-08-30

**Authors:** Bernard Allaouchiche

**Affiliations:** Intensive Care Unit, Centre Hospitalier Lyon Sud-France, Lyon, France

Dear Editor-in-Chief,

I read with interest the excellent study on the effects of selective decontamination in French ICUs [1], which seems to show a reduction in ICU-acquired infections. In particular, the authors report a decrease in the incidence of bacteraemia and pneumonia.

Despite its name, selective digestive decontamination is not always selective. In fact, numerous articles have reported the systemic passage of aminoglycosides.

In Gastinne’s article [2], 82% of patients had tracheal aspirate tobramycin levels greater than 0.18 mg/l.

In the same article, more than 50% of patients had detectable blood tobramycin levels.

Moöhlmann [2, 3] showed in a retrospective study that the median serum tobramycin concentration in patients with SDD was 0.33 mg/l.

In these conditions, the usual microbiological criteria for diagnosing respiratory infections are unreliable, and monitoring of tobramycin serum concentrations may be required in cases of renal failure.

In short, the systemic and intratracheal passage of antibiotics used in SDD makes the diagnosis of infection difficult and the interpretation of comparative results highly questionable.

Prof B. Allaouchiche.

Intensive Care Unit.

Centre Hospitalier Lyon Sud-France

Email : bernard.allaouchiche@gmail.com.

## References

[1] Massart N, Leone M, Reizine F, Duclos G, Machut A, Vacheron CH, Savey A, Hammad E, Friggeri A, Lepape A: Selective decontamination regimens in French ICUs: association with reduced infection and resistance emergence. Ann Intensive Care. 2025:15(1):41. 10.1186/s13613-025-01465-9 PMC11936853 40131603

[2] Gastinne H, Wolff M, Lachatre G, Boiteau R, Savy FP: Antibiotic levels in bronchial tree and in serum during selective digestive decontamination. Intensive Care Med. 1991:17(4):215–218. 10.1007/BF01709880 1744306

[3] Möhlmann JE, van Luin M, Mascini EM, van Leeuwen HJ, de Maat MR: Monitoring of tobramycin serum concentrations in selected critically ill patients receiving selective decontamination of the digestive tract: a retrospective evaluation. Eur J Clin Pharmacol. 2019:75(6):831–836. 10.1007/s00228-019-02644-x 30778624

